# Precedence of Bone Loss Accompanied with Changes in Body Composition and Body Fat Distribution in Patients with Type 2 Diabetes Mellitus

**DOI:** 10.1155/2023/6753403

**Published:** 2023-04-17

**Authors:** Biao Zheng, Yuxin Zheng, Yongze Zhang, Lingning Huang, Ximei Shen, Fengying Zhao, Sunjie Yan

**Affiliations:** ^1^Department of Endocrinology, The First Affiliated Hospital, Fujian Medical University, Fuzhou 350005, China; ^2^Department of Endocrinology, National Regional Medical Center, Binhai Campus of the First Affiliated Hospital, Fujian Medical University, Fuzhou 350212, China; ^3^Clinical Research Center for Metabolic Diseases of Fujian Province, The First Affiliated Hospital, Fujian Medical University, Fuzhou 350005, China; ^4^Fujian Key Laboratory of Glycolipid and Bone Mineral Metabolism, The First Affiliated Hospital, Fujian Medical University, Fuzhou 350005, China; ^5^Diabetes Research Institute of Fujian Province, The First Affiliated Hospital, Fujian Medical University, Fuzhou 350005, China; ^6^Metabolic Diseases Research Institute, The First Affiliated Hospital, Fujian Medical University, Fuzhou 350005, China

## Abstract

**Methods:**

A total of 596 patients with T2DM, including 308 male and 288 female patients, were included in the follow-up study; the median follow-up time was 2.17 years. We calculated the difference between the endpoint and the baseline of each body composition index and the annual rate. The research participants were divided into the increased body mass index (BMI) group, stable BMI group, and decreased BMI group. Some confounding factors were adjusted, such as BMI, fat mass index (FMI), muscle mass index (MMI), muscle/fat mass ratio (M/F), trunk fat mass index (TFMI), appendicular skeletal muscle mass index (ASMI), and appendicular skeletal muscle mass/trunk fat mass ratio (A/T).

**Results:**

The linear analysis showed that *Δ*FMI and *Δ*TFMI were negatively correlated with the change in femoral neck BMD (*Δ*FNBMD) and *Δ*MMI, *Δ*ASMI, *Δ*M/F, and *Δ*A/T were positively correlated with *Δ*FNBMD. The risk of FNBMD reduction in patients with increased BMI was 56.0% lower than that in patients with decreased BMI; also, the risk in patients with stable M/F was 57.7% lower than that in patients with decreased M/F. The risk in the A/T increase group was 62.9% lower than that in the A/T decrease group.

**Conclusions:**

A reasonable muscle/fat ratio is still beneficial to maintaining bone mass. Maintaining a certain BMI value is conducive to maintaining FNBMD. Simultaneously, increasing the proportion of muscle mass and reducing fat accumulation can also prevent FNBMD loss.

## 1. Introduction

Osteoporosis is a systemic skeletal disease characterized by low bone mass and microarchitectural deterioration of bone tissue [1]. It is the leading cause of fragility fractures in elderly people. Weight loss, increasing age, menopause, and type 2 diabetes mellitus (T2DM) are all risk factors for osteoporosis and fragility fractures [1, 2]. Patients with T2DM are at high risk of fractures and hence have received much attention [3]. Early intervention targeting the risk factors for bone loss in T2DM can effectively reduce the incidence of osteoporosis and improve the prognosis [2].

T2DM, which is defined as a progressive loss of adequate *β*-cell insulin secretion frequently on the background of insulin resistance, is associated with inflammation, metabolic stress, and genetic factors [4]. Many patients with T2DM have an imbalance of bone mineral metabolism, and the coexistence of nephropathy tends to aggravate this [5]. Other complications also contribute to fractures. Patients with diabetic peripheral neuropathy have balance and gait problems, increasing the fall risk [3]. People with T2DM often experience weight changes during subsequent visits. Previous studies pointed out that being overweight was a protective factor for bone mineral density (BMD) [6, 7], and a lower body mass index (BMI) was associated with a higher risk of fractures [8]. However, other studies found that weight gain led to a decrease in BMD [9]. Obese individuals had a higher risk of fracture [10], and obesity was not conducive to fracture healing [11]. At present, no unified conclusion exists about the correlation between body weight and BMD loss and even fracture risk.

Body weight includes fat, muscle, bone minerals, and other components. The proportion of fat and muscle is not the same, even if the body weight is the same, due to differences in body composition among individuals. Studies have shown that muscles protect BMD from mechanical and endocrine effects [12]. People with reduced muscle mass and obesity have a higher risk of fractures compared with people who are simply obese [13]. The results of the correlation between fat and bone density in different parts are inconsistent [14, 15], and the association between fat and bone is affected by age, sex, and fat location [16]. The weight change in patients with T2DM is also accompanied by changes in fat, muscle mass and its distribution, and mass composition ratio. Assessing the changes in BMD only by patient weight is clearly inappropriate. Few studies have focused on the relationship between body composition changes and BMD in patients with diabetes during follow-up.

Therefore, this study was conducted to analyze the correlation between body composition changes and BMD in patients with T2DM with different body weight changes and to explore the beneficial body composition parameters for maintaining bone mass.

## 2. Materials and Methods

### 2.1. Research Population

This study was a retrospective cohort study involving 596 patients with T2DM (308 men and 288 women) who were admitted to the Department of Endocrinology, the First Affiliated Hospital of Fujian Medical University, from 2016 to 2020. We did not limit the age of menopause of women to ensure that young women were also selected for the study so as to balance the age gap of the study population. The inclusion criteria were as follows: (1) patients meeting the criteria for the diagnosis of diabetes [17] and (2) patients having complete data on body composition and BMD. The exclusion criteria were as follows: (1) patients with type 1 diabetes, gestational diabetes, other special types of diabetes, diabetic ketoacidosis, hyperosmotic hyperglycemia syndrome, hypoglycemia, and lactic acidosis; (2) patients with acute and chronic infection, hepatic and renal insufficiency, cardiac insufficiency, malnutrition, and cancer; (3) patients who had used or were using antiosteoporotic drugs, such as zoledronic acid, denosumab, or other hypoglycemic drugs that affected BMD, such as thiazolidinediones, sodium glucose cotransporter-2 inhibitors (empagliflozin, dapagliflozin, etc.), dipeptidyl peptidase-4 inhibitors (sitagliptin, etc.), and glucagon-like peptide-1 receptor agonists (such as liraglutide); [3, 18] (4) patients excluding the influence of secondary factors (hyperthyroidism, parathyroid dysfunction, chronic nephropathy, etc.) to exclude other forms of secondary osteoporosis [2]; and (5) athletes or pregnant women.

The serum samples collected from patients in this study showed that the serum vitamin D concentration was 24.16 ± 7.98 ng/ml, and 13.4% of the patients used vitamin D and calcium. Some scholars believe that serum vitamin D in elderly people should be greater than or equal to 30 ng/ml, which could reduce the risk of fracture and prevent osteoporosis [2]. However, some scholars still set this standard to 20 ng/ml [2]. Our research population was distributed in southern China with plenty of sunshine. Therefore, vitamin D deficiency was not serious in the population in this study. To sum up, in terms of mild vitamin D deficiency, the use of vitamin D or calcium in some patients did not significantly affect the final results of the study. This study was approved by the ethics committee of the First Affiliated Hospital of Fujian Medical University (MRCTA, ECFAH of FMU, 2017-131), and the participants provided informed consent.

### 2.2. Sample Size Determination

The sample size was determined for logistic regression using the Power Analysis and Sample Size (PASS) software, version 15.0. The cross-sectional study was planned to have 80% statistical power with a 95% confidence interval (CI) and a case-to-control ratio of nearly 1 : 1. According to the pilot study and previous studies, the probability of BMD reduction was about 70%. The incidence of BMD in this study population was about 60% after preliminary statistics. The estimated sample size was 487 for cases. This study comprised two follow-ups of the same patient, and the control and case groups had the same samples. So, the sample size of this study was 487. The sample size was determined for the exposure status of different variables. The largest sample size among these exposure variables was taken. Allowing a nonresponse rate of 10%, the optimal sample size was 536.

### 2.3. Research Methods

#### 2.3.1. Medical History Collection and Physical Examination

We used the medical history of patients to record the basic data such as age, sex, drinking history, smoking history, T2DM course, other previous medical history, and medication history. We downloaded patient-related medical record data by retrieving the patient's ID number and hospitalization number to reduce the error caused by manual entry and thus to ensure its accuracy and authenticity.

All patients underwent a detailed physical examination, and data on height, weight, blood pressure, and so forth were collected. Height and weight were measured on the RGZ-120-RT scale: The patient stood barefoot, with heels close together and the heels, sacrum, and shoulders close to the post of the altimeter. Systolic blood pressure (SBP) and diastolic blood pressure (DBP) were measured after 15 min of rest. BMI = weight (kg)/height^2^ (m^2^).

#### 2.3.2. BMD and Body Composition Examination

The whole-body fat mass, whole-body muscle mass, trunk fat mass, limb muscle mass, BMD of first to fourth lumbar vertebrae (L_1-4_BMD), and femoral neck BMD (FNBMD) were measured by dual-energy X-ray absorptiometry (DEXA, American GELUNAR company, Prodigy Type). Every year, we calibrate the standard BMD phantom of the instrument, which is made of hydroxyapatite, in our hospital and define the 1.000 g/cm^2^ BMD value of the instrument. All BMD reports were evaluated by professional radiologists to eliminate the effects of osteophytes and other artifacts as much as possible. FMI was calculated using the following formula: whole − body fat mass (kg)/height^2^ (m^2^); MMI was calculated as muscle mass (kg)/height^2^ (m^2^); TFMI was calculated as trunk fat mass (kg)/height^2^ (m^2^); and ASMI was calculated as appendicular skeletal muscle mass (kg)/height^2^ (m^2^). M/F was calculated using the following formula: whole − body muscle mass (kg)/whole − body fat mass (kg); and A/T was calculated using the following formula: limb muscle mass (kg)/trunk fat mass (kg). The change value was obtained from the data at the endpoint of follow-up minus the data at the baseline. The annual change rates were adjusted by follow-up time (year). The intra- and interassay coefficients of variation for DEXA were 0.64% and 0.80%, respectively.

#### 2.3.3. Clinical Biochemical Examination

The blood samples were collected after fasting for more than 8 h. We measured the levels of fasting plasma glucose, glycosylated hemoglobin A1c (HbA1c) (variant II glycosylated hemoglobin analyzer, Bio-Rad, high-performance liquid chromatography), total cholesterol, triglyceride (TG), high-density lipoprotein cholesterol (HDL-C), and low-density lipoprotein cholesterol (LDL-C) (Siemens ADVIA 2400 automatic biochemical analyzer). All intra- and interassay coefficients of variation were 10%.

#### 2.3.4. Statistical Analysis

The research data were statistically analyzed using SPSS Statistics 25.0 software (IBM Corp., NY, USA). The normality test was carried out using the single-sample Kolmogorov-Smirnov test. The quantitative data with normal distribution were expressed as average ± standard deviation, whereas the “median (quartile range)” was used for data with nonnormal distribution. The differences in demographic characteristics, physical examination, and clinical and metabolic parameters of the patients were compared. One-way analysis of variance was used for continuous variables with normal distribution, whereas the rank-sum test was used for continuous variables with nonnormal distribution. Pearson chi-square test (*χ*^2^ test) was used for classified variables. Linear regression and binary logistic regression were used to analyze the correlation between BMI, FMI, MMI, M/F, TFMI, ASMI, A/T, L_1-4_BMD, and FNBMD. The outliers were rechecked and corrected; otherwise, they were deleted. A *P* value < 0.05 indicated a statistically significant difference.

### 2.4. Diagnosis and Definition

#### 2.4.1. Evaluation of Chronic Complications of Diabetes Mellitus

*(1) Diabetic Kidney Disease*. The kidneys of patients with diabetes are often damaged by hyperglycemia, resulting in abnormal intracellular metabolism, leading to microinflammation and subsequent expansion of the extracellular matrix, which gradually develops into nephropathy [19]. Patients often have persistently high levels of albuminuria [20]. Diabetic kidney disease is one of diabetic microvascular complications, which is the leading cause of end-stage renal disease. After excluding kidney disease caused by other nondiabetic factors, the diagnostic criteria of diabetic nephropathy were eGFR < 60 ml/min and/or UACR > 30 mg/g; it lasted for 3 months [20].

*(2) Diabetic Retinopathy*. Whether the patient had or did not have vision loss, we recommended that the patient complete the fundus examination. The fundus examination is performed by professional ophthalmologists after mydriasis. The fundus examination after mydriasis showed typical retinal changes, including microhemangioma, hemorrhage, and exudate [20]. Diabetic retinopathy was classified into different risk groupings.

*(3) Diabetic Peripheral Neuropathy*. Possible symptoms of diabetic peripheral neuropathy include loss of sensation and numbness or prickling or stabbing or burning of the lower limbs; the signs may include a symmetric decrease in distal sensation or unequivocally decreased or absent ankle reflexes [21]. The pain sensation was detected with acupuncture, the touch sensation was examined with a 10 g nylon wire, temperature sensation was detected with a temperature sensation tester, the vibration sensation was tested with a standard 128 Hz tuning fork, and the ankle reflex was examined with a tendon hammer [22]. This study referred to the clinical diagnostic criteria recommended by the Toronto International Conference on Diabetic Peripheral Neuropathy in 2009 [21]: (1) definite history of diabetes; (2) peripheral neuropathy during or after the diagnosis of diabetes; (3) clinical symptoms and signs consistent with diabetic peripheral neuropathy; and (4) for patients with clinical symptoms (pain, numbness, sensory abnormality, etc.), any one of the five examinations (acupuncture pain, tactile pressure, temperature sensation, vibration sensation, and ankle reflex) was abnormal, and for those without clinical symptoms, any two of the five examinations were abnormal, and the patients were clinically diagnosed with diabetic peripheral neuropathy (neuropathy caused by other causes should be excluded at the same time).

### 2.5. Grouping Criteria

We measured BMI, body composition index and its constituent ratio, and FNBMD at the baseline and the endpoint of follow-up. A previous study showed that the BMI increase rate in patients with diabetes was about 0.2 kg/(m^2^ ∙ year^−1^). Therefore, we calculated the change rate of BMI <–0.2 (kg/(m^2^ ∙ year^−1^)), –0.2 (kg/(m^2^ ∙ year^–1^)), and >0.2 (kg/(m^2^ ∙ year^−1^)) and then divided the patients into decreased BMI group, stable BMI group, and increased BMI group [23]. A previous study showed that the leg muscle mass of patients in the intervention group increased by about 3% compared with the leg muscle mass in those without any special intervention under the influence of exercise and diet. With reference to this value, we set the cut-off value at 3%. Therefore, if the body composition change rate exceeded 3%, the patients were classified into the corresponding group. Hence, the indicators were defined as decreasing, stabilizing, and increasing by the percentage changes in FMI, MMI, M/F, TFMI, ASMI, and A/T <–3%, –3 to ~3%, and >3% [24]. Some scholars set the lowest difference in FNBMD change, which was a statistically significant value to evaluate the reduction of fracture risk in patients with diabetes. Therefore, the FNBMD percentage change was defined as <–2%, –2% to 2%, and >2% [25].

## 3. Results

### 3.1. Demographic Analysis

#### 3.1.1. Demographic Characteristics of Patients

**Figure 1:**
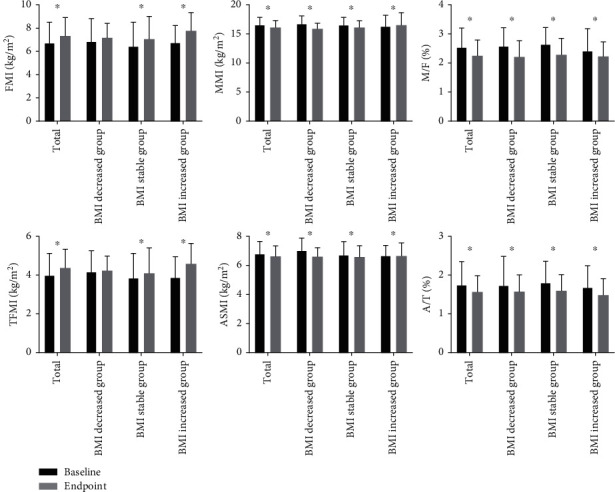
Bar graphs of body composition index and constituent ratio in 596 T2DM patients before and after follow-up. Note: FMI: fat mass index; MMI: muscle mass index; M/F: muscle/fat mass ratio; TFMI: trunk fat mass index; ASMI: appendicular skeletal muscle mass index; A/T: appendicular skeletal muscle mass/trunk fat mass ratio.

As shown in Table 1 and Figure 1, 596 patients with T2DM (308 men and 288 women) were enrolled in this retrospective cohort study. The average baseline age was 60.77 ± 10.86 years, the average course of T2DM was 8.95 ± 6.98 years, and the median follow-up time was 2.17 years.

#### 3.1.2. Demographic Characteristics of Patients with Different BMI Values

As shown in Table 1 and Figure 1, patients with T2DM had no significant change in BMI and L_1-4_BMD at the end of follow-up (*P* > 0.05), but FNBMD, ASMI, M/F, and A/T decreased (*P* > 0.05) and FMI and TFMI increased (*P* < 0.05) compared with the baseline. Furthermore, the patients with T2DM were divided into three groups: decreased BMI group, stable BMI group, and decreased BMI group. At the end of the follow-up, FNBMD, M/F, and A/T decreased in all groups (*P* < 0.05) (Supplemental Table S1).

### 3.2. Linear Analysis Results of *Δ*FMI, *Δ*MMI, *Δ*M/F, *Δ*TFMI, *Δ*ASMI, *Δ*A/T, *Δ*L_1-4_BMD, and *Δ*FNBMD

**Figure 2:**
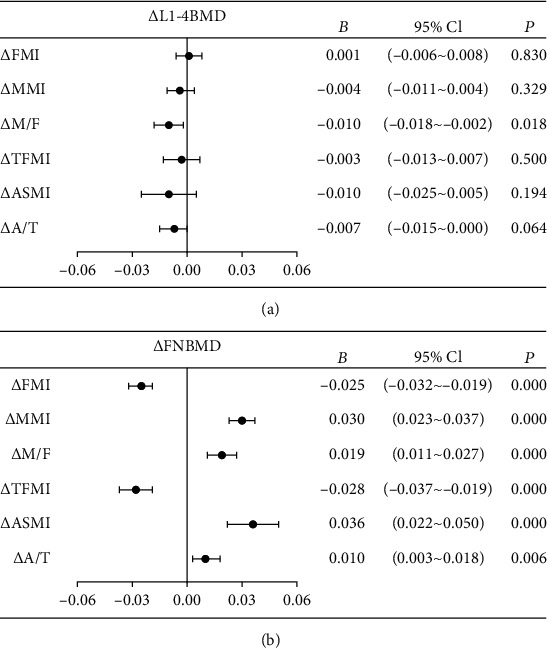
Linear regression analysis of body composition index, L_1-4_BMD, and FNBMD. Note: adjusted: age, sex, course of T2DM, chronic complications of T2DM, BMI, FBG, HbA1c, TG, LDL-C, HDL-C, SBP, DBP, and medication history. FMI: fat mass index; MMI: muscle mass index; M/F: muscle/fat mass ratio; TFMI: trunk fat mass index; ASMI: appendicular skeletal muscle mass index; A/T: appendicular skeletal muscle mass/trunk fat mass ratio.

As shown in Figure 2, almost no statistically significant difference was observed in the body composition index of *Δ*L_1-4_BMD after adjusting for confounding factors (age, sex, course of T2DM, chronic complications of T2DM, BMI, FBG, HbA1c, TG, LDL-C, HDL-C, SBP, DBP, and medication history) in all patients with T2DM; however, a negative correlation was found between *Δ*M/F and *Δ*L_1-4_BMD (*B* = –0.010, 95%Cl = –0.018–0.002, *P* = 0.018), which could explain 0.9% of the variation in *Δ*L_1-4_BMD, and the influence was low (adjusted *R*^2^ = 0.8%). The body composition indices had a significant influence on *Δ*FNBMD. *Δ*FMI and *Δ*TFMI were negatively correlated with *Δ*FNBMD (*Δ*FMI: *B* = –0.025, 95%Cl = –0.032–0.019, *P* < 0.001; *Δ*TFMI: *B* = –0.028, 95%Cl = –0.037–0.019, *P* < 0.001), which could explain 9.1% and 5.5% of the variation in *Δ*FNBMD, respectively. *Δ*MMI, △*Δ*M/F, *Δ*ASMI, and *Δ*A/T were positively correlated with *Δ*FNBMD (*Δ*MMI: *B* = 0.030, 95%Cl = 0.023–0.037, *P* < 0.001; *Δ*M/F: *B* = 0.019, 95%Cl = 0.011–0.027, *P* < 0.001; *Δ*ASMI: *B* = 0.036, 95%Cl = 0.022–0.050, *P* < 0.001; *Δ*A/T: *B* = 0.010, 95%Cl = 0.003–0.018, *P* = 0.006) (Supplemental Table S2.1, S2.2).

### 3.3. Binary Logistic Regression Analysis of BMI, FMI, MMI, M/F, TFMI, ASMI, A/T, and FNBMD

**Figure 3:**
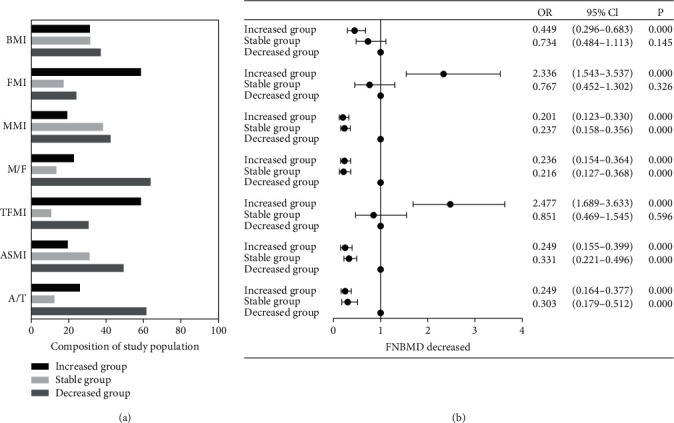
Binary logistic regression analysis of body mass index, body composition index, and FNBMD reduction. Note: adjusted: age, sex, course of T2DM, chronic complications of T2DM, BMI, FBG, HbA1c, TG, LDL-C, HDL-C, SBP, DBP, and medication history. FMI: fat mass index; MMI: muscle mass index; M/F: muscle/fat mass ratio; TFMI: trunk fat mass index; ASMI: appendicular skeletal muscle mass index; A/T: appendicular skeletal muscle mass/trunk fat mass ratio.

As shown in Figure 3, the risk of FNBMD reduction in the increased BMI group was 55.1% lower than that in the reduced BMI group (odds ratio (OR) = 0.449, 95%Cl = 0.296–0.683, *P* < 0.001) after adjusting for confounding factors (age, sex, course of T2DM, chronic complications of T2DM, FBG, HbA1c, TG, LDL-C, HDL-C, SBP, DBP, and medication history). The risk of FNBMD reduction in the increased BMI group and stable group was significantly lower than that in the decreased BMI group (*P* < 0.001), and the same trend was also observed in M/F, ASMI, and A/T (*P* < 0.001). Further, when FMI and TFMI increased, the risk of FNBMD reduction was 2.336 and 2.477 times compared with that in the decreased BMI group (FMI: OR = 2.336, 95%Cl = 1.543–3.537, *P* < 0.001; TFMI: OR = 2.477, 95%Cl = 1.689–3.633, *P* < 0.001). The stable M/F group seemed to have a slight advantage in protecting FNBMD compared with the increased M/F group (Supplemental Table S3.1, S3.2).

## 4. Discussion

Many factors influence BMD in patients with T2DM. Except for common risk factors such as age, prolonged course of T2DM, and increased chronic complications of diabetes [26, 27], body weight is an important factor. Further, the body weight changes with muscle and fat.

The BMD decreases with age, and the average decline in bone loss begins in the fifth decade of life, which ranges from 0.5% to 1.0% every year [2]. Patients with T2DM have pathophysiological conditions such as insulin deficiency, glucose toxicity, accumulation of advanced glycation end products, production of adipose-derived factors, including proinflammatory cytokines and adipokines, and inhibition of Wnt pathway, leading to impairment of mechanical balance, bone turnover, and collagen properties of osteocytes, resulting in the loss of bone mass [28].

Weight changes occur in patients with T2DM during treatment, and most of them are characterized by weight gain. Previous studies showed that increased BMI was a protective factor for BMD, but being overweight or even obese was also an important risk factor for diabetes, cardiovascular disease, and so forth [2, 29]. Further, obese elderly people have an increased risk of falls, degenerative diseases, and systemic diseases. Therefore, people with higher BMI do not have healthy bones [6]. Obesity may affect BMD through the following mechanisms: increasing the mechanical load on bones; producing more estrogen to regulate the activity of osteoblasts and osteoclasts, but enriching vitamin D and testosterone to reduce the serum concentration [30]; and regulating bone strength through some biochemical factors, such as adiponectin and leptin [31, 32], increasing the expression of SOST, DKK1, and FGF23 and inhibiting the expression of Wnt pathway and the function of 1*α*-hydroxylase [30], resulting in the loss of bone mass. Weight loss or emaciation is closely related to osteoporosis [2, 33] and may also increase the risk of cardiovascular events and death in patients with heart failure [34] and coronary heart disease [35, 36]. The risk of death after being infected in elderly people with weight loss is 75% [37]. Therefore, it is not comprehensive to guide the early identification and prevention of risk factors of osteoporosis only through BMI to evaluating the ideal body weight and ignoring its body composition. This study found that the change trend of M/F and A/T was consistent with that of FNBMD, while BMI was not consistent with that of FNBMD. FNBMD decreased in patients with decreased, stable, and increased BMI. And the patients in the increased BMI group were accompanied by the decrease of M/F and A/T. The above results suggest that the factor affecting BMD is body composition or constituent ratio, not BMI.

Compared with BMI, the evaluation of BMD by M/F and A/T covers the favorable effects of muscle and the adverse effects of fat; it also reflects the effect of body composition ratio on BMD. Previous studies showed that the risk of osteoporosis and nonvertebral fractures was more than three times higher in elderly people with muscle loss and obesity than in those who were simply obese [13]. This study found that the risk of FNBMD reduction was more than 50% lower in patients with stable M/F than in patients with decreased M/F, and was more than 60% lower in patients with increased A/T than in patients with decreased A/T. It suggested that the increase in M/F and A/T had a protective effect on FNBMD. The underlying mechanism was that the adipose tissue could inhibit osteogenesis [38], whereas the mechanical load and cytokine stimulation of muscle had beneficial effects on bone [39–41]. Therefore, the increase in muscle/lipid ratio might be beneficial to the bone. However, the data on the direct relationship between muscle/fat ratio and BMD are lacking, demanding further basic experiments and large-sample follow-up work.

This study found that the increase in FMI and TFMI was a risk factor for the loss of FNBMD, whereas the increase in MMI and ASMI was beneficial to FNBMD. People with a high percentage of body fat had a significantly higher risk of osteoporosis and fracture [42], and the decrease in fat mass caused by weight loss was beneficial to bone health [14]. Further, studies showed that osteoblasts and adipocytes originated from common stromal cells. *In vitro*, bone mass loss was related to the increase in the amount of adipose tissue in bone marrow [43]. The study by Akune et al. [38] and other studies confirmed that the peroxisome proliferator–activated receptor pathway was the main regulatory pathway of adipogenesis, which could inhibit osteoblast differentiation. Therefore, excessive fat accumulation is harmful for BMD. As an endocrine organ, muscle plays a beneficial role in bone by secreting cytokines such as interleukin-6 [41]. At the same time, muscle protects bone by means of mechanical stress. Aging leads to a decrease in the mechanical movement of muscle and bone, resulting in an age-related decrease in bone strength with reduced physical activity [39, 40]. For every 1 standard deviation of muscle mass, the incidence of osteoporosis decreases by 30% [44, 45].

Site-specific differences were found in the effects of fat and muscle mass on BMD. Kang et al. [15] found that the increase in trunk fat mass was positively correlated with spinal bone mineral density, which was beneficial to local lumbar BMD. Also, some studies showed that visceral fat and spinal bone marrow fat accumulation were disadvantageous to spinal BMD and reduced abdominal fat was good for bone health [14]. This study found that the change in the fat index did not change L_1-4_BMD because the study comprised a few people with obvious abdominal obesity. Thus, the typical data to explore the effect of abdominal fat on L_1-4_BMD were lacking. The whole-body and trunk fat measured in this study included different types of fat, such as subcutaneous and visceral fat. Different types and parts of fat might have different effects on lumbar BMD. Compared with abdominal fat, lower limb fat is an ideal fat repository and plays a protective role in systemic metabolic processes such as bone metabolism [16]. The limb muscle is the most important exercise and weight-bearing muscle in the whole body. The mechanical stress of limb muscle stimulates the femur and other limb bones [41]. A significant positive correlation was observed between the decrease in limb muscle index and osteoporosis. The risk of osteoporosis in patients with sarcopenia was 12.7 times higher than that in patients without sarcopenia [46–48].

In this study, no significant change in L_1-4_BMD was found before and after follow-up, suggesting that body weight and body composition had no significant effect on L_1-4_BMD. The possible reasons were as follows. First, the muscles stimulated the bones of the femur and other extremities mainly through compression, bending, and torsion. In contrast, the axial bones of the lumbar vertebrae were stimulated by axial compression [41, 49]. Therefore, the mechanical action suffered by the axial bone was relatively small, and hence, the muscle had no significant effect on the BMD of the lumbar vertebrae. Second, in this study, the use of DEXA to measure lumbar BMD was extremely affected by abdominal fat, visceral fat, and abdominal subcutaneous tissue. Although the BMD reports were approved by professional radiologists during measurement, they might have measurement errors.

In summary, we should comprehensively consider patients' age, course of T2DM, chronic complications of diabetes, fat mass, muscle mass, muscle fat ratio, and other factors to evaluate the fracture risk of patients with T2DM. Appropriately maintaining M/F and A/T can slow down bone loss and reduce the incidence of osteoporosis and the risk of brittle fracture to some extent.

This study had the following limitations and shortcomings. First, the cases in this study came from patients in the endocrinology department. Although each BMD report was evaluated by professional radiologists, errors existed in the interpretation of the results, leading to hospitalization bias. Second, some confounding factors, such as past history, medical experience, and menopausal age of women, could not be adjusted due to the limitations of computational power and the actual situation. Third, the patients were divided into groups based on the annual change rate of BMI, but the BMI span of the participants at baseline was still large. Therefore, data with a larger sample size were needed for subgroup analysis.

## 5. Conclusions

Although age and disease duration are important risk factors for bone loss in patients with diabetes and differences exist in the effects of muscle and fat on BMD, a reasonable muscle/fat ratio is still beneficial to the maintenance of bone mass. Maintaining a certain BMI value is conducive to maintaining FNBMD. Simultaneously, increasing the proportion of muscle mass and reducing fat accumulation can also prevent FNBMD loss.

## Figures and Tables

**Table 1:** Demographic data of 596 patients with T2DM.

	Total			Decreased BMI group			Stable BMI group			Increased BMI group		
	Baseline	Endpoint	*P*	Baseline	Endpoint	*P*	Baseline	Endpoint	*P*	Baseline	Endpoint	*P*
*N*	596	596	/	221	221	/	188	188	/	187	187	/
Age (yr)	61 (53-68)	64 (56-70)	<0.001^∗^	62 (53-69)	64 (55-71)	<0.001^∗^	63 (54-70)	65 (56-72)	<0.001^∗^	61 (53-68)	63 (54-70)	<0.001^∗^
Male, *n* (%)	308 (51.68)	/	/	114 (51.6)	/	/	104 (55.3)	/	/	90 (48.1)	/	/
T2DM course (yr)	8.00 (3.00-13.00)	10.58 (5.33-14.75)	<0.001^∗^	8.00 (3.00-13.50)	10.08 (5.08-15.71)	<0.001^∗^	8.50 (3.00-13.00)	10.58 (5.27-14.67)	<0.001^∗^	9.00 (4.00-12.00)	10.92 (5.83-14.42)	<0.001^∗^
DR, *n* (%)	106 (17.79)	160 (26.85)	<0.001^∗^	28 (12.67)	42 (19.00)	<0.001^∗^	31 (16.49)	56 (29.79)	<0.001^∗^	47 (25.13)	62 (33.16)	<0.001^∗^
DPN, *n* (%)	262 (43.96)	322 (54.03)	<0.001^∗^	83 (37.56)	115 (52.04)	<0.001^∗^	90 (47.87)	100 (53.19)	<0.001^∗^	89 (47.59)	107 (57.22)	<0.001^∗^
DKD, *n* (%)	107 (17.95)	164 (27.52)	<0.001^∗^	37 (16.74)	56 (25.34)	<0.001^∗^	34 (18.09)	51 (27.13)	<0.001^∗^	36 (19.25)	57 (30.48)	<0.001^∗^
HT, *n* (%)	313 (52.52)	/	/	120 (54.3)	/	/	100 (53.2)	/	/	93 (49.7)	/	/
Smoking, *n* (%)	96 (16.11)	/	/	36 (16.3)	/	/	36 (19.1)	/	/	24 (12.8)	/	/
Drinking, *n* (%)	147 (24.66)	/	/	10 (4.5)	/	/	8 (4.3)	/	/	129 (68.6)	/	/
SBP (mmHg)	136 (122-150)	135 (125-149)	0.264	137 (123-150)	133 (121-149)	0.150	136 (120-148)	134 (124-148)	0.353	135 (124-149)	139 (128-153)	0.005^∗^
DBP (mmHg)	78 (70-84)	78 (71-85)	0.762	79 (70-84)	78 (70-85)	0.402	79 ± 11	77 ± 10	0.071	78 ± 10	77 ± 11	0.178
FPG (mmol/l)	7.72 (5.87-11.11)	7.08 (5.25-9.84)	<0.001^∗^	7.62 (5.84-10.68)	6.80 (5.38-9.49)	0.003^∗^	7.72 (5.90-11.47)	7.24 (5.11-10.29)	0.018^∗^	8.21 (5.91-11.15)	7.22 (5.30-11.50)	0.001^∗^
HbA1c (%)	8.66 (7.10-10.81)	7.90 (6.70-9.70)	<0.001^∗^	8.40 (7.00-10.20)	7.60 (6.50-9.75)	0.007^∗^	8.30 (7.10-10.81)	7.90 (6.80-9.80)	0.001^∗^	9.40 (7.60-11.50)	8.00 (6.70-9.30)	<0.001^∗^
TC (mmol/l)	4.60 (3.85-5.38)	4.29 (3.54-5.08)	<0.001^∗^	4.64 ± 1.15	4.31 ± 1.19	<0.001^∗^	4.62 (3.76-5.30)	4.34 (3.57-5.12)	0.025^∗^	4.66 (3.96-5.42)	4.47 ± 1.16	0.001^∗^
TG (mmol/l)	1.44 (0.95-2.16)	1.35 (0.98-2.05)	0.481	1.45 (0.98-2.18)	1.27 (0.92-1.93)	0.007^∗^	1.41 (0.93-2.12)	1.39 (1.00-2.14)	0.732	1.42 (0.94-2.16)	1.47 (1.00-2.28)	0.216
HDL-C (mmol/l)	1.12 (0.92-1.34)	1.03 (0.87-1.27)	<0.001^∗^	1.10 (0.94-1.30)	1.03 (0.89-1.24)	0.003^∗^	1.16 (0.94-1.34)	1.01 (0.87-1.25)	<0.001^∗^	1.18 ± 0.39	1.11 ± 0.36	0.002^∗^
LDL-C (mmol/l)	2.83 (2.14-3.45)	2.64 (1.94-3.31)	0.001^∗^	2.84 ± 1.01	2.67 ± 1.01	0.005^∗^	2.77 ± 0.92	2.77 ± 1.00	0.973	2.85 (2.26-3.53)	2.67 (2.01-3.36)	0.004^∗^
Metformin, *n* (%)	370 (62.08)	423 (70.97)	<0.001^∗^	147 (66.52)	167 (75.57)	<0.001^∗^	114 (60.64)	126 (67.02)	<0.001^∗^	109 (58.29)	129 (69.52)	<0.001^∗^
Insulin, *n* (%)	215 (36.07)	315 (52.85)	<0.001^∗^	71 (32.13)	95 (42.99)	<0.001^∗^	66 (35.11)	107 (56.91)	<0.001^∗^	78 (41.71)	113 (60.43)	<0.001
L_1-4_BMD (g/cm^2^)	1.06 (0.92-1.17)	1.05 (0.93-1.18)	0.223	1.06 (0.93-1.16)	1.06 (0.93-1.16)	0.190	1.05 (0.90-1.18)	1.04 (0.92-1.20)	0.060	1.05 (0.93-1.16)	1.06 (0.94-1.18)	0.031^∗^
FNBMD (g/cm^2^)	0.92 (0.80-1.03)	0.86 (0.75-0.96)	<0.001^∗^	0.93 (0.79-1.09)	0.86 (0.74-0.98)	<0.001^∗^	0.91 (0.80-0.99)	0.84 (0.77-0.93)	<0.001^∗^	0.91 (0.83-1.00)	0.87 (0.77-0.97)	<0.001^∗^

Note: BMI: body mass index; T2DM: type 2 diabetes mellitus; DR: diabetic retinopathy; DPN: diabetic peripheral neuritis; DKD: diabetic kidney disease; HT: hypertension; SBP: systolic blood pressure; DBP: diastolic blood pressure; FPG: fasting plasma glucose; HbA1c: glycated hemoglobin; TC: total cholesterol; TG: triglycerides; HDL-C: high-density lipoprotein cholesterol; LDL-C: low-density lipoprotein cholesterol. ^∗^*P* < 0.05.

## Data Availability

The data used and/or analyzed during the current study are available from the corresponding author on reasonable request.

## Abbreviations

BMD: Bone mineral density
T2DM: Type 2 diabetes mellitus
BMI: Body mass index
FMI: Fat mass index
MMI: Muscle mass index
M/F: Muscle/fat mass ratio
TFMI: Trunk fat mass index
ASMI: Appendicular skeletal muscle mass index
A/T: Appendicular skeletal muscle mass/trunk fat mass ratio
FNBMD: Femoral neck bone mineral density
L_1-4_BMD: Lumbar 1st-4th bone mineral density
SGLT-2: Sodium glucose cotransporter-2
GLP-1: Glucagon-like peptide-1
SBP: Systolic blood pressure
DBP: Diastolic blood pressure
FPG: Fasting blood glucose
DEXA: Dual-energy X-ray absorptiometry
HbA1c: Glycosylated hemoglobin A1c
TC: Total cholesterol
TG: Triglyceride
HDL-C: High-density lipoprotein cholesterol
LDL-C: Low-density lipoprotein cholesterol
DKD: Diabetic kidney disease
DR: Diabetic retinopathy
DPN: Diabetic peripheral neuropathy
AGEs: Advanced glycation end products
PPAR: Peroxisome proliferator-activated receptor.

## Supplementary Materials

Supplementary Materials Table S1: body composition index and constituent ratio in 596 T2DM patients before and after follow-up. Table S2.1: linear regression analysis of body composition index and L1-4BMD. Table S2.2: linear regression analysis of body composition index and FNBMD. Table S3.1: frequency of body mass index and body composition index. Table S3.2: binary logistic regression analysis of body mass index, body composition index, and FNBMD reduction.
