# Supplementary material for: Precedence of Bone Loss Accompanied with Changes in Body Composition and Body Fat Distribution in Patients with Type 2 Diabetes Mellitus
Source: J Diabetes Res. 2023 Apr 17;2023:6753403. doi: 10.1155/2023/6753403 (PMC10125744; doi:10.1155/2023/6753403)
Supplement: Supplementary Materials — Table S1: body composition index and constituent ratio in 596 T2DM patients before and after follow-up. Table S2.1: linear regression analysis of body composition index and L1-4BMD. Table S2.2: linear regression analysis of body composition index and FNBMD. Table S3.1: frequency of body mass index and body composition index. Table S3.2: binary logistic regression analysis of body mass index, body composition index, and FNBMD reduction. [file 6753403.f1.zip › Supplementary Table (1) Body composition index and constituent ratio (1).docx]

**Table S1 Body composition index and constituent ratio in 596 T2DM patients before and after follow-up**

|  | Total | | | | BMI decreased group | | | | BMI stable group | | | | BMI increased group | | | |
| --- | --- | --- | --- | --- | --- | --- | --- | --- | --- | --- | --- | --- | --- | --- | --- | --- |
|  | Baseline | Endpoint | Z(T) | P | Baseline | Endpoint | Z(T) | P | Baseline | Endpoint | Z(T) | P | Baseline | Endpoint | Z(T) | P |
| N | 596 | 596 | / | / | 221 | 221 | / | / | 188 | 188 | / | / | 187 | 187 | / |  |
| FMI  (kg/m^2^) | 6.72(5.15-8.51) | 7.32(5.85-8.91) | -10.914 | <0.001 | 6.85(5.06-8.83) | 7.16(5.91-8.41) | -0.681 | 0.496 | 6.44(5.28-8.51) | 7.04(5.72-9.00) | -7.755 | <0.001 | 6.75(5.14-8.22) | 7.76(6.23-9.34) | -9.929 | <0.001 |
| MMI  (kg/m^2^) | 16.55(15.22-17.85) | 16.09(14.92-17.29) | -8.529 | <0.001 | 16.74(15.66-18.09) | 15.88(14.50-16.85) | -11.378 | <0.001 | 16.53(14.94-17.85) | 16.09(15.10-17.29) | -4.298 | <0.001 | 16.30±1.92 | 16.51±2.12 | -2.997 | 0.003 |
| M/F  (%) | 2.54(1.87-3.20) | 2.25(1.74-2.79) | -13.190 | <0.001 | 2.58(1.84-3.21) | 2.20(1.81-2.77) | -6.829 | <0.001 | 2.64(1.86-3.22) | 2.28(1.72-2.85) | -7.221 | <0.001 | 2.41(1.89-3.18) | 2.22(1.65-2.72) | -8.699 | <0.001 |
| TFMI  (kg/m^2^) | 3.99(2.99-5.12) | 4.36(3.47-5.34) | -9.274 | <0.001 | 4.18(3.11-5.26) | 4.23(3.44-4.98) | -1.063 | 0.228 | 3.85(2.91-5.12) | 4.09(3.47-5.40) | -6.860 | <0.001 | 3.88(2.91-4.95) | 4.58(3.68-5.63) | -9.492 | <0.001 |
| ASMI  (kg/m^2^) | 6.79(6.07-7.65) | 6.62(5.86-7.34) | -8.651 | <0.001 | 7.02(6.24-7.88) | 6.60(5.65-7.21) | -11.962 | <0.001 | 6.72(6.06-7.63) | 6.57(6.03-7.35) | -3.523 | <0.001 | 6.67(5.81-7.38) | 6.64(6.00-7.54) | -4.284 | <0.001 |
| A/T  (%) | 1.74(1.32-2.35) | 1.56(1.19-1.98) | -12.014 | <0.001 | 1.73(1.28-2.48) | 1.57(1.20-2.00) | -5.225 | <0.001 | 1.80(1.33-2.36) | 1.59(1.19-2.01) | -6.791 | <0.001 | 1.68(1.30-2.24) | 1.48(1.16-1.91) | -8.476 | <0.001 |
