# Supplementary material for: Precedence of Bone Loss Accompanied with Changes in Body Composition and Body Fat Distribution in Patients with Type 2 Diabetes Mellitus
Source: J Diabetes Res. 2023 Apr 17;2023:6753403. doi: 10.1155/2023/6753403 (PMC10125744; doi:10.1155/2023/6753403)
Supplement: Supplementary Materials — Table S1: body composition index and constituent ratio in 596 T2DM patients before and after follow-up. Table S2.1: linear regression analysis of body composition index and L1-4BMD. Table S2.2: linear regression analysis of body composition index and FNBMD. Table S3.1: frequency of body mass index and body composition index. Table S3.2: binary logistic regression analysis of body mass index, body composition index, and FNBMD reduction. [file 6753403.f1.zip › Supplementary Table (2.1) body composition Index and L1-4BMD (1).docx]

**Table S2.1 Linear regression Analysis of body composition Index and L_1-4_BMD**

| Cat. | B | β | t | Sig. | 95%Cl |
| --- | --- | --- | --- | --- | --- |
| ΔFMI  (kg/m^2^) | 0.001 | 0.009 | 0.215 | 0.83 | -0.006~0.008 |
| ΔMMI  (kg/m^2^) | -0.004 | -0.04 | -0.976 | 0.329 | -0.011~0.004 |
| ΔM/F  (%) | -0.010 | -0.097 | -2.382 | 0.018 | -0.018~-0.002 |
| ΔTFMI  (kg/m^2^) | -0.003 | -0.028 | -0.675 | 0.500 | -0.013~0.007 |
| ΔASMI  (kg/m^2^) | -0.010 | -0.053 | -1.301 | 0.194 | -0.025~0.005 |
| ΔA/T  (%) | -0.007 | -0.076 | -1.855 | 0.064 | -0.015~0.000 |

* Adjusted for confounding factors: age, sex, course of T2DM, chronic complications of T2DM, BMI, FBG, HbA1c, TG, LDL-C, HDL-C, SBP, DBP and medication history.
