# Supplementary material for: Precedence of Bone Loss Accompanied with Changes in Body Composition and Body Fat Distribution in Patients with Type 2 Diabetes Mellitus
Source: J Diabetes Res. 2023 Apr 17;2023:6753403. doi: 10.1155/2023/6753403 (PMC10125744; doi:10.1155/2023/6753403)
Supplement: Supplementary Materials — Table S1: body composition index and constituent ratio in 596 T2DM patients before and after follow-up. Table S2.1: linear regression analysis of body composition index and L1-4BMD. Table S2.2: linear regression analysis of body composition index and FNBMD. Table S3.1: frequency of body mass index and body composition index. Table S3.2: binary logistic regression analysis of body mass index, body composition index, and FNBMD reduction. [file 6753403.f1.zip › Supplementary Table (2.2) body composition Index and FNBMD (1).docx]

**Table S2.2 Linear regression Analysis of body composition Index and FNBMD**

| Cat. | B | β | t | Sig. | 95%Cl |
| --- | --- | --- | --- | --- | --- |
| ΔFMI  (kg/m^2^) | -0.025 | -0.302 | -7.709 | <0.001 | -0.032~-0.019 |
| ΔMMI  (kg/m^2^) | 0.030 | 0.339 | 8.711 | <0.001 | 0.023~0.037 |
| ΔM/F  (%) | 0.019 | 0.195 | 4.844 | <0.001 | 0.011~0.027 |
| ΔTFMI  (kg/m^2^) | -0.028 | -0.235 | -5.891 | <0.001 | -0.037~-0.019 |
| ΔASMI  (kg/m^2^) | 0.036 | 0.198 | 4.931 | <0.001 | 0.022~0.050 |
| ΔA/T  (%) | 0.010 | 0.111 | 2.733 | 0.006 | 0.003~0.018 |

* Adjusted for confounding factors: age, sex, course of T2DM, chronic complications of T2DM, BMI, FBG, HbA1c, TG, LDL-C, HDL-C, SBP, DBP and medication history.
