# Supplementary material for: Precedence of Bone Loss Accompanied with Changes in Body Composition and Body Fat Distribution in Patients with Type 2 Diabetes Mellitus
Source: J Diabetes Res. 2023 Apr 17;2023:6753403. doi: 10.1155/2023/6753403 (PMC10125744; doi:10.1155/2023/6753403)
Supplement: Supplementary Materials — Table S1: body composition index and constituent ratio in 596 T2DM patients before and after follow-up. Table S2.1: linear regression analysis of body composition index and L1-4BMD. Table S2.2: linear regression analysis of body composition index and FNBMD. Table S3.1: frequency of body mass index and body composition index. Table S3.2: binary logistic regression analysis of body mass index, body composition index, and FNBMD reduction. [file 6753403.f1.zip › Supplementary Table (3.1) Frequency of body mass index, body composition index (1).docx]

**Table S3.1 Frequency of body mass index, body composition index**

|  | Cat. | Frequency | Rate (%) |
| --- | --- | --- | --- |
| BMI | Increased group | 187 | 31.4 |
|  | Stable group | 188 | 31.5 |
|  | Decreased group | 221 | 37.1 |
| FMI | Increased group | 349 | 58.6 |
|  | Stable group | 103 | 17.3 |
|  | Decreased group | 144 | 24.2 |
| MMI | Increased group | 115 | 19.3 |
|  | Stable group | 228 | 38.3 |
|  | Decreased group | 253 | 42.4 |
| M/F | Increased group | 136 | 22.8 |
|  | Stable group | 80 | 13.4 |
|  | Decreased group | 380 | 63.8 |
| TFMI | Increased group | 349 | 58.6 |
|  | Stable group | 64 | 10.7 |
|  | Decreased group | 183 | 30.7 |
| ASMI | Increased group | 116 | 19.5 |
|  | Stable group | 186 | 31.2 |
|  | Decreased group | 294 | 49.3 |
| A/T | Increased group | 155 | 26 |
|  | Stable group | 74 | 12.4 |
|  | Decreased group | 367 | 61.6 |
