# Supplementary material for: Precedence of Bone Loss Accompanied with Changes in Body Composition and Body Fat Distribution in Patients with Type 2 Diabetes Mellitus
Source: J Diabetes Res. 2023 Apr 17;2023:6753403. doi: 10.1155/2023/6753403 (PMC10125744; doi:10.1155/2023/6753403)
Supplement: Supplementary Materials — Table S1: body composition index and constituent ratio in 596 T2DM patients before and after follow-up. Table S2.1: linear regression analysis of body composition index and L1-4BMD. Table S2.2: linear regression analysis of body composition index and FNBMD. Table S3.1: frequency of body mass index and body composition index. Table S3.2: binary logistic regression analysis of body mass index, body composition index, and FNBMD reduction. [file 6753403.f1.zip › Supplementary Table (3.2) Body mass index, body composition index and FNBMD reduction (1).docx]

**Table S3.2 Binary logistic regression analysis of body mass index, body composition index and FNBMD reduction**

| Cat. | OR | 95%Cl | P |
| --- | --- | --- | --- |
| BMI |  |  | 0.001 |
| -Increased group | 0.449 | 0.296~0.683 | <0.001 |
| -Stable group | 0.734 | 0.484~1.113 | 0.145 |
| -Decreased group | 1 | / | / |
| FMI |  |  | <0.001 |
| -Increased group |  | 2.336 | 1.543~3.537 |
| -Stable group | 0.326 | 0.767 | 0.452~1.302 |
| -Decreased group | 1 | / | / |
| MMI |  |  | <0.001 |
| -Increased group | 0.201 | 0.123~0.330 | <0.001 |
| -Stable group | 0.237 | 0.158~0.356 | <0.001 |
| -Decreased group | 1 | / | / |
| M/F |  |  | <0.001 |
| -Increased group | 0.236 | 0.154~0.364 | <0.001 |
| -Stable group | 0.216 | 0.127~0.368 | <0.001 |
| -Decreased group | 1 | / | / |
| TFMI |  |  | <0.001 |
| -Increased group | 2.477 | 1.689~3.633 | <0.001 |
| -Stable group | 0.851 | 0.469~1.545 | 0.596 |
| -Decreased group | 1 | / | / |
| ASMI |  |  | <0.001 |
| -Increased group | 0.249 | 0.155~0.399 | <0.001 |
| -Stable group | 0.331 | 0.221~0.496 | <0.001 |
| -Decreased group | 1 | / | / |
| A/T |  |  | <0.001 |
| -Increased group | 0.249 | 0.164~0.377 | <0.001 |
| -Stable group | 0.303 | 0.179~0.512 | <0.001 |
| -Decreased group | 1 | / | / |

* Adjusted for confounding factors: age, sex, course of T2DM, chronic complications of T2DM, BMI, FBG, HbA1c, TG, LDL-C, HDL-C, SBP, DBP and medication history.
